# Dietary Zinc Deficiency Exaggerates Ethanol-Induced Liver Injury in Mice: Involvement of Intrahepatic and Extrahepatic Factors

**DOI:** 10.1371/journal.pone.0076522

**Published:** 2013-10-14

**Authors:** Wei Zhong, Yantao Zhao, Xinguo Sun, Zhenyuan Song, Craig J. McClain, Zhanxiang Zhou

**Affiliations:** 1 Center for Translational Biomedical Research, University of North Carolina at Greensboro, North Carolina Research Campus, Kannapolis, North Carolina, United States of America; 2 Department of Nutrition, University of North Carolina at Greensboro, North Carolina Research Campus, Kannapolis, North Carolina, United States of America; 3 Department of Medicine, University of Louisville, Louisville, Kentucky, United States of America; 4 Department of Pharmacology & Toxicology, University of Louisville, Louisville, Kentucky, United States of America; 5 Robley Rex Louisville VAMC, Louisville, Kentucky, United States of America; 6 Department of Kinesiology and Nutrition, University of Illinois at Chicago, Chicago, Illinois, United States of America; State University of Rio de Janeiro, Biomedical Center, Institute of Biology, Brazil

## Abstract

Clinical studies have demonstrated that alcoholics have a lower dietary zinc intake compared to health controls. The present study was undertaken to determine the interaction between dietary zinc deficiency and ethanol consumption in the pathogenesis of alcoholic liver disease. C57BL/6N mice were subjected to 8-week feeding of 4 experimental liquid diets: (1) zinc adequate diet, (2) zinc adequate diet plus ethanol, (3) zinc deficient diet, and (4) zinc deficient diet plus ethanol. Ethanol exposure with adequate dietary zinc resulted in liver damage as indicated by elevated plasma alanine aminotransferase level and increased hepatic lipid accumulation and inflammatory cell infiltration. Dietary zinc deficiency alone increased hepatic lipid contents, but did not induce hepatic inflammation. Dietary zinc deficiency showed synergistic effects on ethanol-induced liver damage. Dietary zinc deficiency exaggerated ethanol effects on hepatic genes related to lipid metabolism and inflammatory response. Dietary zinc deficiency worsened ethanol-induced imbalance between hepatic pro-oxidant and antioxidant enzymes and hepatic expression of cell death receptors. Dietary zinc deficiency exaggerated ethanol-induced reduction of plasma leptin, although it did not affect ethanol-induced reduction of white adipose tissue mass. Dietary zinc deficiency also deteriorated ethanol-induced gut permeability increase and plasma endotoxin elevation. These results demonstrate, for the first time, that dietary zinc deficiency is a risk factor in alcoholic liver disease, and multiple intrahepatic and extrahepatic factors may mediate the detrimental effects of zinc deficiency.

## Introduction

Chronic alcohol consumption leads to alcoholic liver disease, which may evolve through three progressive stages: steatosis, hepatitis, and cirrhosis. Mechanistic studies have suggested that oxidative stress is a basic cellular disorder in the pathogenesis of alcoholic liver disease [1], [2]. Liver is the major organ responsible for ethanol metabolism. Ethanol is first catabolized to acetaldehyde mainly by alcohol dehydrogenase (ADH) and cytochrome P450 2E1 (CYP2E1). Chronic alcohol consumption has been shown to induce hepatic expression of CYP2E1 rather than ADH [3], [4]. CYP2E1 induction has been suggested to be a major mechanism of ethanol-induced oxidative stress in the liver [5]. Other pro-oxidant enzymes such as NADPH oxidase are also involved in ethanol-induced generation of oxidative stress [6]. On the other hand, chronic ethanol consumption reduces hepatic antioxidant enzymes such as superoxide dismutase 1 (SOD-1, also called copper zinc SOD) [4]. Modification and inactivation of cellular proteins under oxidative stress account for ethanol-induced metabolic disorders in the liver [1].

Increasing evidence suggests that chronic alcohol consumption also affects other organ systems such as white adipose tissue (WAT) [7], [8] and intestine [9], [10], and it generates factors which impact the pathogenesis of alcoholic liver disease [11], [12]. Recent studies have shown that WAT dysfunction contributes to the development of alcoholic fatty liver [7], [13], [14]. Chronic alcohol exposure increased fatty acid release from WAT due to hyper-lipolysis, leading to an increased hepatic fatty acid uptake and deposition as triglycerides [7], [14]. Chronic alcohol exposure also reduced WAT secretion of adiponectin and leptin which negatively regulate hepatic lipid contents [15]–[17]. Endotoxemia is a feature in the initiation and progression of inflammation and alcoholic hepatitis. While bacteria overgrowth has been detected in alcoholic liver disease [18], increased gut permeability plays a key role in the development of endotoxemia because endotoxin penetration from the gut lumen to the blood will be limited under conditions of a normal intestinal barrier [19]. Clinical study has revealed that endotoxemia is a crucial factor in the development of alcoholic liver disease because only alcoholics with increased gut permeability developed liver disease [20], [21].

Zinc is an abundant trace element and involves in all the major aspects of cell function, including metabolism, detoxification, antioxidant defense, signaling transduction and gene regulation. Zinc deficiency has been well documented in alcoholic liver disease [22], [23]. Clinical studies have shown that the zinc levels in serum and liver were reduced [22], [24]. Animal studies demonstrated that dietary zinc supplementation attenuates alcohol-induced liver injury [25], [26]. Alcoholics have been shown to have a lower dietary zinc intake from food compared to normal controls [27]. A clinical study showed that alcoholic patients have an average daily zinc intake of 10–11 mg/kg in comparison of 14 mg/kg in healthy controls [28]. The Western world’s shift from consumption of meat proteins to cereal proteins containing high levels of fibers known as phyrates may reduce intestinal zinc absorption [29], [30]. Moreover, the very prevalent use of proton pump inhibitor medications may also create a state of zinc deficiency [31], which makes the issue of zinc deficiency in alcoholic patients more severe. However, the mechanism of how dietary zinc deficiency may impact alcohol-induced liver pathogenesis remains unclear. The present study was designed to determine the interactions of marginal dietary zinc deficiency and chronic alcohol exposure in induction of liver injury, and the involvements of intrahepatic and extrahepatic factors were investigated for a better understanding of the pathogenesis of alcohol-induced liver injury.

## Materials and Methods

### Animals and Treatments

Male C57BL/6N mice were obtained from Harlan (Indianapolis, IN). The animal protocol was approved by the Institutional Animal Care and Use Committee of the North Carolina Research Campus (Permit Number: 10–011). The mice were divided into 4 dietary groups for 8 weeks of feeding: (1) zinc adequate diet (ZnA), (2) zinc adequate diet plus ethanol (ZnA/E), (3) zinc deficient diet (ZnD), and (4) zinc deficient diet plus ethanol (ZnD/E) (n = 8 for ZnA or ZnD group, n = 10 for ZnA/E or ZnD/E group). The liquid diets were prepared according to the Lieber-DeCarli formula with some modifications. The zinc-containing ingredients, casein and mineral mixture, in the original formula were replaced with egg white and zinc-free mineral mixture, respectively. The dietary zinc levels were adjusted to ZnA or ZnD by adding zinc sulfate to the basal diets. For preparing ZnA diets, zinc sulfate was added to the liquid diet to achieve a final concentration of 7.5 ppm elemental zinc (33 mg zinc sulfate/L), while ZnD diets were prepared by adding zinc sulfate to a final concentration of 1.5 ppm elemental zinc (7 mg zinc sulfate/L). For chronic alcohol exposure, mice were pair-fed the Lieber-DeCarli ethanol or isocaloric maltose dextrin control liquid diet with a stepwise feeding procedure as described previously [7]. The ethanol content (%, w/v) in the diet was 4.8 (34% of total calories) for the first 2 weeks, and increased by 0.2% every 2 weeks, reaching 5.4 (38% of total calories) for the last 2 weeks. The amount of food given to the pair-fed mice was that the ethanol-fed mice consumed in the previous day. All ingredients were purchased from Dyets (Bethlehem, PA), and deionized water was used for preparation of the liquid diets. At the end of 8-week feeding, mice were anesthetized with inhalational isoflurane, and blood, liver, intestine and epididymal WAT (eWAT) were collected.

### Blood Metabolites Assays

Blood samples were drawn from the dorsal vena cava and plasma was obtained by centrifuging the blood at 8,000×g for 15 minutes at 4°C. Plasma alanine aminotransferase (ALT) activity was colorimetrically measured using Infinity ALT Reagent provided by Thermo Scientific (Waltham, MA). Plasma keratinocyte chemoattractant (KC) levels were determined with an enzyme-linked immunosorbent assay (ELISA) kit from R&D Systems (Minneapolis, MN). Plasma triglyceride and cholesterol concentrations were determined by Infinity Triglyceride Reagent and Infinity Cholesterol Reagent (Thermo Scientific), respectively. Plasma endotoxin levels were tested using the limulus ameobocyte lysate (LAL) method (Lonza, Walkersville, MD). Plasma leptin was measured using a commercial ELISA kit (Millipore, Billerica, MA). Plasma ethanol concentrations were measured using an ethanol assay kit (BioVision, Milpitas, CA).

### Hepatic Zinc Concentrations

Zinc levels in the liver were determined by atomic absorbance spectrophotometry. The concentrations of zinc were calculated as µg/g dry liver.

### Assessment of Liver Injury

Liver tissues were fixed with 10% formalin and processed for paraffin embedding. Tissue sections were cut at 5 µm and stained with hematoxylin and eosin (H&E) for assessing histopathological changes. The hepatic steatosis was analyzed with the estimation of the proportion of liver parenchyma replaced by lipid droplets using Image Pro Premier (Media Cybernetics, Rockville, MD). For each individual mouse, all 4 liver lobes were included in the assay; at least five non-consecutive random digital images were obtained per liver lobe. The degree of steatosis was quantified as follows: minor (<5%), mild (5%–33%), moderate (34%–66%) or severe (>66%) [32], [33].

For examining inflammation, neutrophils were stained by immunohistochemistry. In brief, liver sections were rehydrated and incubated overnight at 4°C with a polyclonal rat anti mouse Ly-6G antibody (BD Pharmingen, San Jose, CA), followed by incubation with horseradish peroxidase (HRP) labeled goat anti-rat IgG (Thermo Scientific) for 30 minutes. Visualization was conducted using diaminobenzidine (DAKO, Carpinteria, CA) as the HRP substrate. Positive staining of neutrophils in the liver sections were then quantified with Image Pro Premier.

### Determination of Hepatic Lipid Accumulation

Hepatic lipid accumulation was assessed by histochemical detection of neutral lipids and biochemical assay of lipid concentrations. For histochemical determination of neutral lipids, liver tissues were frozen in Tissue-Tek CRYO-OCT compound (Fisher Scientific) and cryostat tissue sections were cut at 7 µm, fixed with 10% formalin for 10 minutes, and stained with an Oil red O procedure. The slides were observed under light microscope, and images were quantified using Image Pro Premier. Hepatic lipids were extracted using chloroform/methanol (2∶1, v/v). Protein in the homogenate was assayed using protein assay reagent (Bio-Rad, Hercules, CA) to normalize the amount of lipid extracted. Triglyceride, cholesterol and free fatty acids (FFAs) levels were measured using assay kits from BioVision.

### qPCR of Hepatic Genes

Total RNA from liver was isolated using TRIzol reagent (Life Technologies, Grand Island, NY) according to the manufacturer’s instructions. The isolated RNA was then reverse transcribed with the TaqMan Reverse Transcription Reagents (Life Technologies) after assessing RNA quantity. Semi-quantitative analysis of relative gene expressions were performed on the Applied Biosystems 7500 Real Time PCR Systems (Applied Biosystems, Carlsbad, CA) using SYBR green PCR Master Mix (Qiagen, Valencia, CA). Primers were designed and synthesized by Integreated DNA Technologies (Coralville, CA) and sequences are listed in Table 1. All primer pairs were validated by demonstrating high amplification efficiency, consistent single peak melt curve and the presence of single product of the expected amplicon size on agarose gel. The relative gene expression was normalized to18s rRNA expression, and calculated using the 2^−ΔΔCt^ method setting the values of ZnA as one.

**Table 1:** Primer sequences used for qPCR analysis.

Gene	Accession No.	Forward/Reverse (5′–3′)	Amplicon Size
KC/Cxcl1	NM_008176	AACCGAAGTCATAGCCACAC/CAGACGGTGCCATCAGAG	118 bp
MCP-1/Ccl2	NM_011333	GTCCCTGTCATGCTTCTGG/GCTCTCCAGCCTACTCATTG	125 bp
IP-10/Cxcl10	NM_021274	TCAGCACCATGAACCCAAG/CTATGGCCCTCATTCTCACTG	132 bp
MIP-1/Ccl3	NM_011337	GATTCCACGCCAATTCATCG/TTCAGTTCCAGGTCAGTGATG	120 bp
TNF-α/Tnfa	NM_013693	CTTCTGTCTACTGAACTTCGGG/CAGGCTTGTCACTCGAATTTTG	150 bp
IL-1β/Il-1b	NM_008361	ACGGACCCCAAAAGATGAAG/TTCTCCACAGCCACAATGAG	143 bp
Cd36	NM_007643	ATGGGCTGTGATCGGAACTG/GTCTTCCCAATAAGCATGTCTCC	110 bp
Fatp2	NM_011978	TCCTCCAAGATGTGCGGTACT/TAGGTGAGCGTCTCGTCTCG	166 bp
Fatp5	NM_009512	CTACGCTGGCTGCATATAGATG/CCACAAAGGTCTCTGGAGGAT	103 bp
Fabp1	NM_017399	ATGAACTTCTCCGGCAAGTACC/CTGACACCCCCTTGATGTCC	118 bp
Acsl1	NM_007981	TGCCAGAGCTGATTGACATTC/GGCATACCAGAAGGTGGTGAG	101 bp
Acc	NM_133360	CTTCCTGACAAACGAGTCTGG/CTGCCGAAACATCTCTGGGA	232 bp
Fas	NM_007988	GGAGGTGGTGATAGCCGGTAT/TGGGTAATCCATAGAGCCCAG	140 bp
Scd1	NM_009127	TTCTTGCGATACACTCTGGTGC/CGGGATTGAATGTTCTTGTCGT	98 bp
Cpt1a	NM_013495	CTCCGCCTGAGCCATGAAG/CACCAGTGATGATGCCATTCT	100 bp
Acadl	NM_007381	TCTTTTCCTCGGAGCATGACA/GACCTCTCTACTCACTTCTCCAG	113 bp
Acox1	NM_015729	TCCAGACTTCCAACATGAGGA/CTGGGCGTAGGTGCCAATTA	286 bp
Cyp4a	NM_010011	TTCCCTGATGGACGCTCTTTA/GCAAACCTGGAAGGGTCAAAC	126 bp
Mttp	NM_008642	CTCTTGGCAGTGCTTTTTCTCT/GAGCTTGTATAGCCGCTCATT	102 bp
Apob	NM_009693	TTGGCAAACTGCATAGCATCC/TCAAATTGGGACTCTCCTTTAGC	142 bp
Dgat1	NM_010046	TCCGTCCAGGGTGGTAGTG/TGAACAAAGAATCTTGCAGACGA	199 bp
Dgat2	NM_026384	GCGCTACTTCCGAGACTACTT/GGGCCTTATGCCAGGAAACT	172 bp
Gpat1	NM_008149	ACAGTTGGCACAATAGACGTTT/CCTTCCATTTCAGTGTTGCAGA	139 bp
Lipc	NM_008280	ATGGGAAATCCCCTCCAAATCT/GTGCTGAGGTCTGAGACGA	207 bp
Ppara	NM_011144	AGAGCCCCATCTGTCCTCTC/ACTGGTAGTCTGCAAAACCAAA	153 bp
Hnf1a	NM_009327	GACCTGACCGAGTTGCCTAAT/CCGGCTCTTTCAGAATGGGT	103 bp
Hnf4a	NM_008261	GCCTTCTGCGAACTCCTTCTG/GGGACGATGTAGTCATTGCCT	137 bp
Leprb	NM_146146	TGGTCCCAGCAGCTATGGT/ACCCAGAGAAGTTAGCACTGT	102 bp
18s rRNA	NR_003278	GTAACCCGTTGAACCCCATT/CCATCCAATCGGTAGTAGCG	151 bp

### Measurements of Hepatic Lipid Peroxidation

The lipid peroxidation products were measured by thiobarbituric acid reactive substances (TBARS) method using a commercial kit (Cayman Chemical, Ann Arbor, MI). Hepatic 4-hydroxynonenal (4-HNE) and malondialdehyde (MDA) levels were detected by immunohistochemistry. Briefly, liver tissue sections were treated with 3% hydrogen peroxide for 10 minutes to inactivate endogenous peroxidases. The endogenous mouse IgG was blocked by incubation with a mouse-to-mouse blocking reagent (ScyTek Laboratories, Logan, UT). Tissue sections were then incubated with a monoclonal mouse anti-4-HNE antibody or a monoclonal mouse anti-MDA antibody (Northwest Life Science Specialties, Vancouver, WA) at 4°C overnight, followed by incubation with EnVision^+^ Labled Polymer-HRP-conjugated anti-mouse IgG (DAKO, Carpinteria, CA) for 30 minutes. Diaminobenzidine (DAKO) was used as HRP substrate for visualization. The negative controls were conducted by omitting the primary antibody.

### Immunoblot Analysis

Whole protein lysates of liver tissue were extracted using 10% Nonidet P-40 lysis buffer supplemented with 1% protease inhibitor cocktail and 1% phenylmethylsulfonyl fluoride. Protein concentrations were measured with a protein assay reagent based on the Bradford method (Bio-Rad). Aliquots containing 60 µg total proteins were loaded onto a 8%–12% sodium dodecyl sulfate-polyacrylamide gel, transblotted onto polyvinylidene difluoride (PVDF) membrane (Bio-Rad), blocked with 5% nonfat dry milk in Tris-buffered saline with 0.1% Tween-20, and then incubated with each of the primary antibodies which are listed in Table 2, including CYP2E1, p47^phox^, inducible nitric oxide synthase (iNOS), superoxide dismutase-1 (SOD-1), SOD-2, glutathione peroxidase (GPx 1/2), thioredoxin (Trx), thioredoxin reductase 1 (TrxR1), tumor necrosis factor receptor 1 (TNFR1), Fas/CD95, and β-actin. The membrane was then incubated with horseradish peroxidase-conjugated donkey anti-rabbit or goat anti-mouse IgG (Thermo Scientific). The bound complexes were detected with enhanced chemiluminescence (GE Healthcare, Piscataway, NJ) and quantified by densitometry analysis.

**Table 2:** Primary antibodies for immunoblot analysis and immunohistochemistry.

Primary Antibody	Full Name	Molecular wt.	Source	Maker
CYP2E1	Cytochrome P450 2E1	56 kDa	Rabbit	Abcam
p47^phox^	NADPH oxidase subunit (47 kDa)	47 kDa	Goat	Santa Cruz Biotechnology
iNOS	Inducible nitric oxide synthase	130 kDa	Rabbit	Santa Cruz Biotechnology
SOD-1	Superoxide dismutase 1	23 kDa	Rabbit	Santa Cruz Biotechnology
SOD-2	Superoxide dismutase 2	24 kDa	Rabbit	Millipore
GPx1/2	Glutathione peroxidase 1/2	23 kDa	Rabbit	Santa Cruz Biotechnology
Trx	Thioredoxin	12 kDa	Rabbit	Santa Cruz Biotechnology
TrxR1	Thioredoxin receptor 1	55 kDa	Rabbit	Santa Cruz Biotechnology
TNFR1	Tumor necrosis factor receptor 1	55 kDa	Rabbit	Santa Cruz Biotechnology
CD95	Cluster of differentiation 95; Fas	48 kDa	Rabbit	Santa Cruz Biotechnology
β-Actin	Beta-actin	42 kDa	Mouse	Sigma
Ly-6G	Lymphocyte antigen 6 complex, locus G	21–25 kDa	Mouse	BD Biosciences
4-HNE	4-Hydroxynonenal	–	Mouse	Northwest Life Science Specialties
MDA	Malondialdehyde	–	Mouse	Northwest Life Science Specialties

### Histopathological Examination of eWAT

The abdominal eWAT was fixed in 10% formalin, and processed for paraffin embedding. Paraffin tissue sections were cut at 7 µm, and stained with H&E staining.

### Gut Permeability Assay

For *ex vivo* detection of intestinal permeability, the ileum was freshly isolated and placed in a modified Krebs-Henseleit bicarbonate buffer containing 8.4 mM HEPES, 119 mM NaCl, 4.7 mM KCl, 1.2 mM MgSO_4_, 1.2 mM KH_2_PO_4_, 25 mM NaHCO_3_, 2.5 mM CaCl_2_ and 11 mM glucose (KHBB, pH 7.4). One end of the gut segment was first ligated with suture, and 100 µl FITC-dextran (M.W. 4,000, FD-4, 40 mg/ml) was injected into the lumen using a gavage needle to avoid mucosal injury. Then the other end of the gut segment was ligated to form an 8-cm gut sac. After rising in the KHBB buffer, the gut sac was placed in 2 ml of KHBB and incubated at 37°C for 20 minutes. The FD-4 that penetrated from the lumen into the incubation buffer was measured spectrofluorometrically with an excitation wave length of 485 nm and an emission wave length of 530 nm. The FD-4 permeability was expressed as µg/min/cm.

### Statistics

Data are expressed as mean ± standard deviation (SD). The results were analyzed using analysis of variance (ANOVA) followed by Newman-Keuls’ multiple comparison test. In all tests, *P* values less than 0.05 were considered statistically significant.

## Results

### Body Weight, Liver Weight, eWAT Weight and Plasma Lipid Levels

As shown in Table 3, ethanol feeding reduced body weight regardless of dietary zinc status. The liver weight of ZnA/E group was not significantly different from that of ZnA group. ZnD group showed an increased liver weight compared to ZnA, while the liver weight of ZnD/E group was greater than both ZnA and ZnA/E groups. The liver/body weight ratio was increased in ZnA/E group compared to ZnA group, while ZnD/E group showed a further increase with a value which was higher than all the other 3 groups. On the other hand, ethanol feeding reduced the eWAT weight and eWAT/body weight ratio regardless of dietary zinc status. Ethanol feeding with adequate zinc did not affect plasma triglyceride level, while ethanol feeding with dietary zinc deficiency increased the plasma triglyceride level. Ethanol feeding decreased plasma cholesterol levels both in ZnA/E and ZnD/E groups.The plasma ethanol concentrations after 8 weeks of ethanol consumption were 142.04±31.23 mg/dL for ZnA/E group and 146.45±27.17 mg/dL for ZnD/E group (*P*>0.05), respectively. Ethanol feeding decreased zinc concentrations in the liver (84.60±14.32 µg/g for ZnA/E group versus 130.70±15.61 µg/g for ZnA group, *P* = 0.009). Dietary zinc deficiency alone decreased hepatic zinc levels (92.12±3.38 µg/g for ZnD group, *P* = 0.014 versus ZnA), and it further decreased hepatic zinc in ethanol feeding mice (56.00±9.59 µg/g for ZnD/E group, *P* = 0.016 versus ZnA/E).

**Table 3:** Body weight, liver weight, and blood parameters of mice fed liquid diets for 8 weeks.

Measurements	ZnA	ZnA/E	ZnD	ZnD/E
Body weight (g)	32.09±0.62^a^	26.70±1.65^b^	32.65±1.17^a^	27.83±1.36^b^
Liver weight (g)	1.27±0.09^a^	1.36±0.10^ab^	1.47±0.10^bc^	1.55±0.18^c^
Liver/body weight ratio (%)	3.97±0.31^a^	4.85±0.30^b^	4.51±0.22^a^	5.76±0.40^c^
eWAT weight (g)	1.51±0.30^a^	0.66±0.09^b^	1.30±0.22^a^	0.55±0.17^b^
eWAT/body weight ratio (%)	4.70±0.91^a^	2.43±0.35^b^	3.97±0.63^a^	1.97±0.53^b^
Plasma triglyceride (mg/dL)	117.28±9.47^a^	123.96±46.66^a^	1112.83±36.99^a^	1154.94±26.81^b^
Plasma cholesterol (mg/dL)	131.30±29.91^a^	84.33±12.20^b^	95.64±23.88^ab^	73.04±18.07^b^
Plasma ethanol (mg/dL)	NA	142.04±31.23	NA	1146.45±27.17
Hepatic zinc (µg/g liver)	130.70±15.61^a^	84.60±14.32^b^	92.12±3.38^b^	56.00±9.59^c^

Data are means ± SD (*n = *8–10). Means with different letters differ at *P*<0.05. NA: not applicable. ZnA: zinc adequate diet. ZnA/E: zinc adequate diet plus ethanol. ZnD: zinc deficient diet. ZnD/E, zinc deficient diet plus ethanol. eWAT: epididymal white adipose tissue.

### Liver Histopathology, Plasma Liver Injury Marker and Inflammatory Cytokine

**Figure 1:**
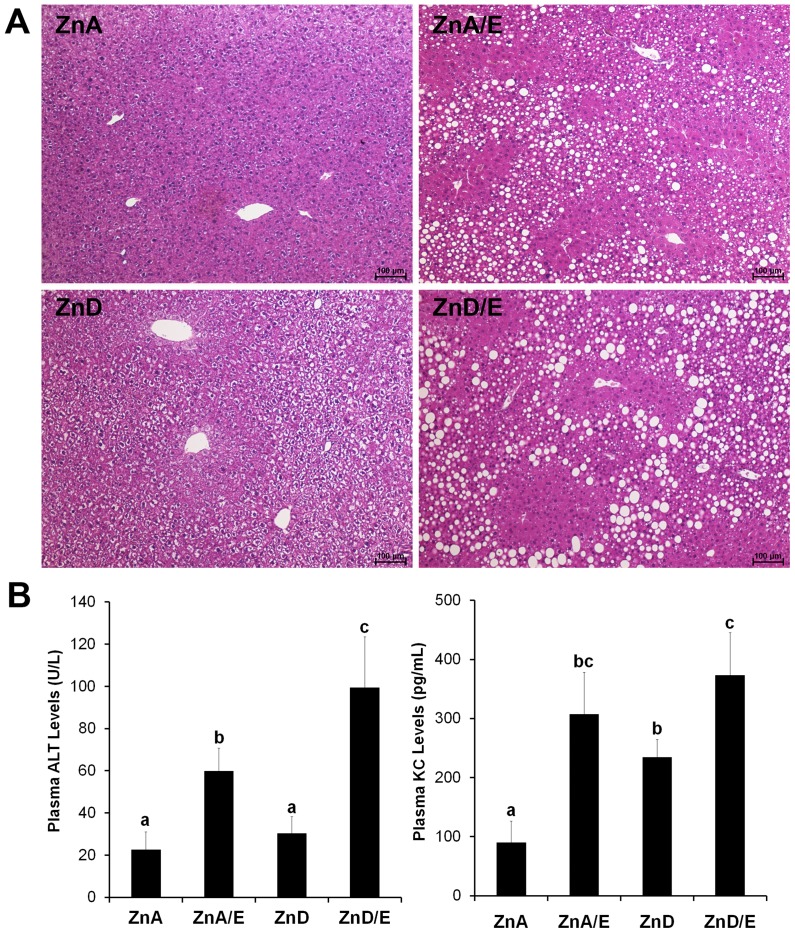
Liver histopathology and plasma markers of liver injury and inflammation in mice chronically fed ethanol with zinc adequate or zinc deficient diet for 8 weeks. **A:** Liver histopathology. H&E staining. CV: central vein. PV: portal vein. Scale bar100 µm. **B:** Plasma alanine aminotransferase (ALT) activities and keratinocyte chemoattractant (KC) levels. Plasma ALT activities were measured using Infinity ALT Reagent. Plasma KC levels were determined with an enzyme-linked immunosorbent assay (ELISA) kit. Results are means ± SD (n8–10). Significant differences (*P*<0.05, ANOVA) are identified by different letters. ZnA: zinc adequate diet. ZnA/E: zinc adequate diet plus ethanol. ZnD: zinc deficient diet. ZnD/E: zinc deficient diet plus ethanol.

As shown in Figure 1A, the most remarkable alteration shown by H&E staining was formation of vacuoles in the liver after ethanol feeding. Although round shape vacuoles were found in the liver of ethanol feeding groups, the sizes were greater in ZnD/E group compared to ZnA/E group. ZnD alone also caused formation of vacuoles, but the size was smaller and the shape was irregular. The degree of steatosis in the liver sections was quantified, and the results were shown in Table 4. Steatosis scores of moderate and severe were more often found in ZnA/E and ZnD/E groups with higher values in the latter. The levels of the plasma liver injury marker, ALT, and the inflammatory cytokine, KC (mouse IL-8 analog), are shown in Figure 1B. ZnA/E group showed an elevated plasma ALT activity compared to ZnA group, while a further elevation was found in ZnD/E group. Plasma KC level was increased in ZnA/E group compared to ZnA group. While zinc deficiency alone also increased plasma KC, ZnD/E group showed a further increase compared to ZnD.

**Table 4:** Quantitative analysis of hepatic steatosis by H&E staining.

Steatosis grade	ZnA (n = 160)	ZnA/E (n = 200)	ZnD (n = 160)	ZnD/E (n = 200)
Minor - <5%	69 (43.1%)	33 (16.5%)	32 (20.0%)	17 (8.5%)
Mild - 5–33%	84 (52.5%)	75 (37.5%)	103 (64.4%)	55 (27.5%)
Moderate - 34–66%	7 (4.4%)	51 (25.5%)	22 (13.7%)	68 (34.0%)
Severe - >66%	0 (0%)	41 (20.5%)	3 (1.9%)	60 (30.0%)

ZnA: zinc adequate diet. ZnA/E: zinc adequate diet plus ethanol. ZnD: zinc deficient diet. ZnD/E: zinc deficient diet plus ethanol. “n” stands for total images analyzed in each group.

### Hepatic Lipid Accumulation and Expression of Genes Related to Lipid Metabolism

**Figure 2:**
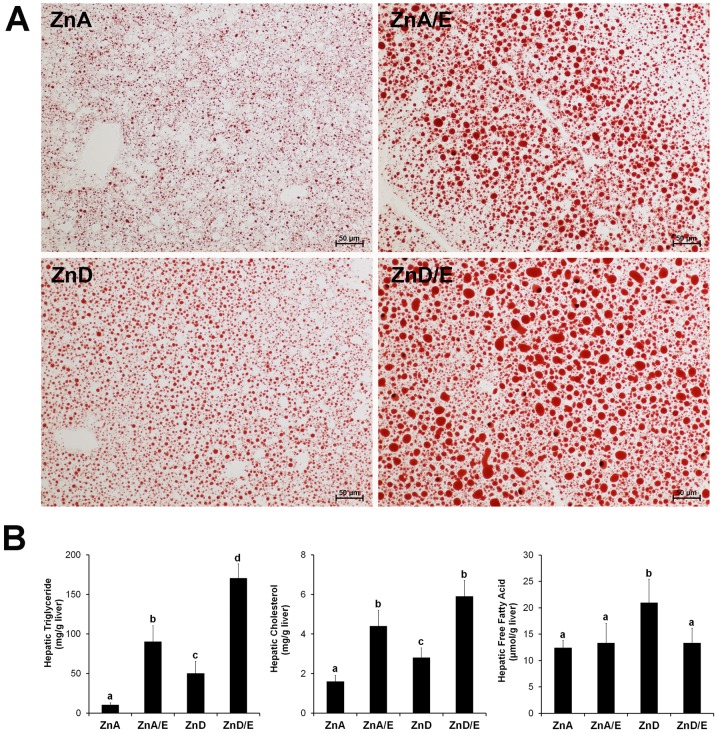
Lipid accumulation in the livers of mice chronically fed ethanol with zinc adequate or zinc deficient diet for 8 weeks. A: Neutral lipid accumulation in the liver. Neutral lipids were stained with Oil red O staining. Scale bar50 µm. **B:** Hepatic lipid concentrations. Triglyceride, cholesterol and free fatty acid were measured using assay kits. Results are means ± SD (n8–10). Significant differences (*P*<0.05, ANOVA) are identified by different letters. ZnA: zinc adequate diet. ZnA/E: zinc adequate diet plus ethanol. ZnD: zinc deficient diet. ZnD/E: zinc deficient diet plus ethanol.

Hepatic lipid accumulation was assessed by Oil red O staining of neutral lipids and quantitative analysis of hepatic triglyceride, cholesterol and FFAs. As shown in Figure 2A, ethanol feeding induced remarkable accumulation of lipid droplets even with adequate zinc (average diameter = 2.74±0.16 µm for ZnA group and 4.46±0.21 µm for ZnA/E group, *P*<0.001). Zinc deficiency alone also caused accumulation of lipid droplets, but the size was relatively smaller (average diameter = 3.59±0.28 µm, *P = *0.002 versus ZnA). Accumulation of many large-sized lipid droplets was found in the liver of ZnD/E group (average diameter = 4.62±0.05 µm, *P* = 0.017 versus ZnA/E). Hepatic concentrations of triglyceride, cholesterol and FFAs are shown in Figure 2B. The triglyceride concentration was significantly higher in ZnA/E group than ZnA group. ZnD group also showed an increased triglyceride level compared to ZnA group, but the level was lower than ZnA/E group. The highest triglyceride level was found in ZnD/E group, a further 2-fold increase compared to ZnA/E group. Hepatic cholesterol level was higher in ZnA/E, ZnD and ZnD/E groups than ZnA, but the level in ZnD group was relatively lower than ZnA/E and ZnD/E groups. Hepatic FFA level was increased only in ZnD group.

**Figure 3:**
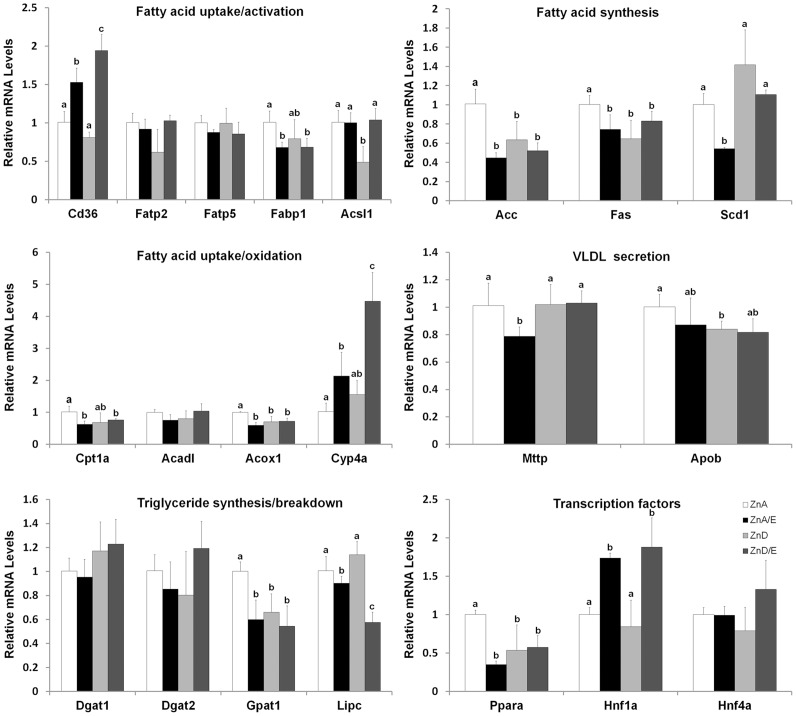
Hepatic expression of genes related to lipid metabolism in mice chronically fed ethanol with zinc adequate or zinc deficient diet for 8 weeks. qPCR analysis was conducted using SYBR green PCR mix. The relative gene expression was normalized to18s rRNA expression, and calculated using the 2^−ΔΔCt^ method setting the values of ZnA as one. Results are means ± SD (n6). Significant differences (*P*<0.05, ANOVA) are identified by different letters. ZnA: zinc adequate diet. ZnA/E: zinc adequate diet plus ethanol. ZnD: zinc deficient diet. ZnD/E: zinc deficient diet plus ethanol.

Figure 3 shows hepatic expression of genes involved in lipid metabolism. Among genes related to fatty acid uptake (Cd36, Fatp2, Fatp5) and transport (Fabp1), Cd36 was up-regulated in ZnA/E and ZnD/E groups with a relatively higher level in ZnD/E group; while Fabp1 was down-regulated in ZnA/E and ZnD/E groups. Fatty acid activation gene (Acsl1) was down-regulated only in the ZnD group, which was correlated well with the increased hepatic FFA concentration. Among the 3 genes related to fatty acid synthesis, Acc and Fas were down-regulated in ZnA/E, ZnD and ZnD/E groups, while Scd1 was down-regulated in ZnD group, compared to ZnA group. Among the 4 genes related to fatty acid oxidation, mitochondrial Cpt1a was down-regulated, but endoplasmic reticulum Cyp4a was up-regulated, in ZnA/E and ZnD/E groups. Peroxisomal Acox1 was down-regulated in ZnA/E, ZnD and ZnD/E groups compared to ZnA group. Among genes related to very low density lipoprotein (VLDL) secretion, down-regulation of Mttp and Apob was found in ZnA/E group and ZnD group, respectively. The triglyceride synthesis gene, Gpat1, was down-regulated in ZnA/E, ZnD and ZnD/E groups compared to ZnA group, while the triglyceride breakdown gene, Lipc, was down-regulated in ZnA/E and ZnD/E groups with a relatively lower value in the latter. A major lipid metabolism regulator gene, Ppara, was down-regulated in ZnA/E, ZnD and ZnD/E groups compared to ZnA group. However, a liver-enriched transcription factor gene, Hnf1a, was up-regulated in ZnA/E and ZnD/E groups.

### Hepatic Infiltration of Neutrophils and Expression of Inflammatory Cytokines

**Figure 4:**
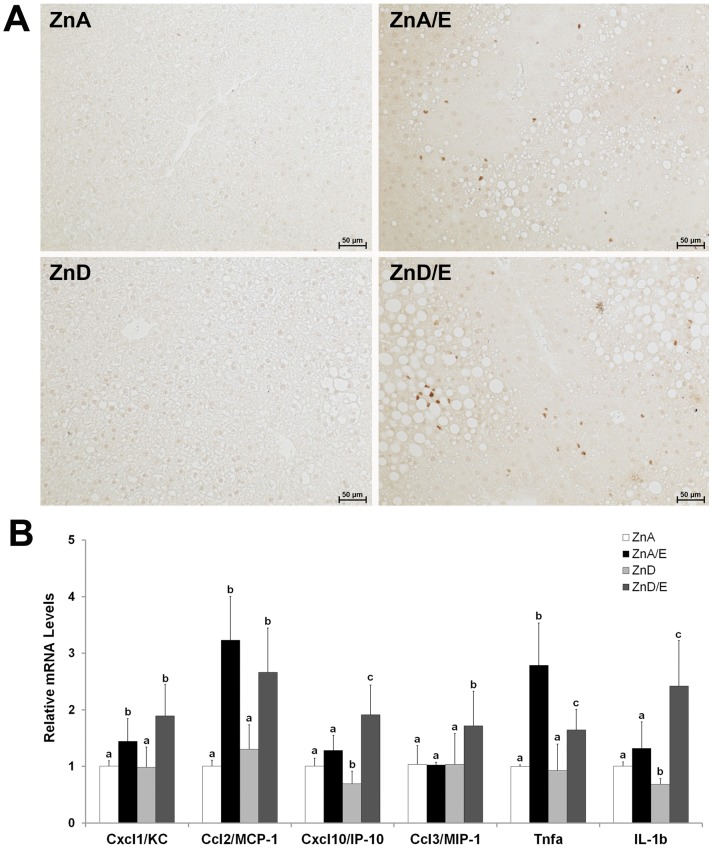
Neutrophil infiltration and inflammatory cytokine gene expression in the livers of mice chronically fed ethanol with zinc adequate or zinc deficient diet for 8 weeks. **A:** Hepatic neutrophil infiltration. Neutrophils were stained by immunohistochemistry with anti-mouse Ly-6G antibody. Scale bar50 µm. **B:** Gene expression of hepatic inflammatory cytokines. qPCR analysis was conducted using SYBR green PCR mix. The relative gene expression was normalized to18s rRNA expression, and calculated using the 2^−ΔΔCt^ method setting the values of ZnA as one. Results are means ± SD (n6). Significant differences (*P*<0.05, ANOVA) are identified by different letters. ZnA: zinc adequate diet. ZnA/E: zinc adequate diet plus ethanol. ZnD: zinc deficient diet. ZnD/E: zinc deficient diet plus ethanol.

Neutrophils were detected by immunohistochemistry and results are shown in Figure 4A. Ethanol feeding with either zinc adequate or zinc deficient diet caused neutrophil infiltration in the liver, but a relatively greater number of neutrophils were found in ZnD/E group (70.67±14.83 positive cells/mm^2^ for ZnA/E group versus 101.86±19.60 positive cells/mm^2^ for ZnD/E group, *P* = 0.007). Zinc deficiency alone did not attract neutrophils to the liver. Figure 4B shows hepatic expression of inflammatory genes. Ethanol feeding with either zinc adequate or zinc deficient diet up-regulated KC, MCP-1 and Tnfa genes, though ZnD/E group showed a relatively less up-regulation of Tnfa compared to ZnA/E. IP-10, MIP-1 and IL-1b genes were up-regulated only in ZnD/E group. Zinc deficiency alone down-regulated IP-10 and IL-1b genes.

### Hepatic Oxidative Stress, Pro-oxidant and Antioxidant Proteins and Cell Death Receptors

**Figure 5:**
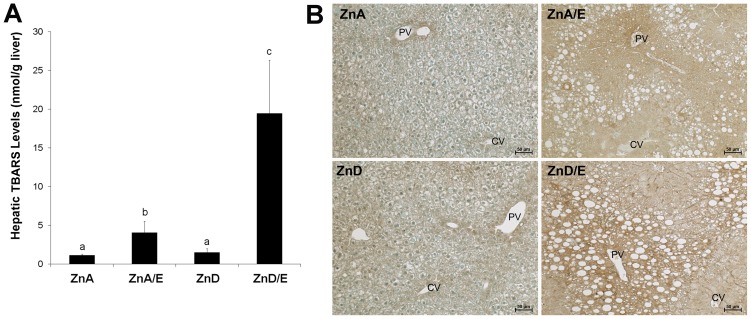
Lipid peroxidation in the livers of mice chronically fed ethanol with zinc adequate or zinc deficient diet for 8 weeks. **A:** Hepatic thiobarbituric acid reactive substances (TBARS) levels. TBARS was measured using a commercial kit. Results are means ± SD (n8–10). Significant differences (*P*<0.05, ANOVA) are identified by different letters. **B:** Hepatic 4-hydroxynonenal (4-HNE) accumulation. Tissue distribution of 4-HNE was detected by immunohistochemistry. CV: central vein. PV: portal vein. Scale car50 µm. ZnA: zinc adequate diet. ZnA/E: zinc adequate diet plus ethanol. ZnD: zinc deficient diet. ZnD/E: zinc deficient diet plus ethanol.

Hepatic oxidative stress was assessed by measuring TBARS levels as well as 4-HNE and MDA generation. As shown in Figure 5A, hepatic TBARS level was increased in both ZnA/E and ZnD/E groups, however, the TBARS level in ZnD/E group was 5-fold greater than that of ZnA/E group. Zinc deficiency alone did not affect hepatic TBARS level. Figure 5B shows the immunohistochemical staining of 4-HNE in the liver. While ZnA/E group exhibited positive staining, ZnD/E group showed more intensive staining compared to ZnA/E group. A relatively stronger staining was found in the area around the portal vein, i.e. zone I. Zinc deficiency alone did not increase hepatic 4-HNE generation. The immunohistochemical staining pattern of MDA was similar to 4-HNE (Supplement Figure S1).

**Figure 6:**
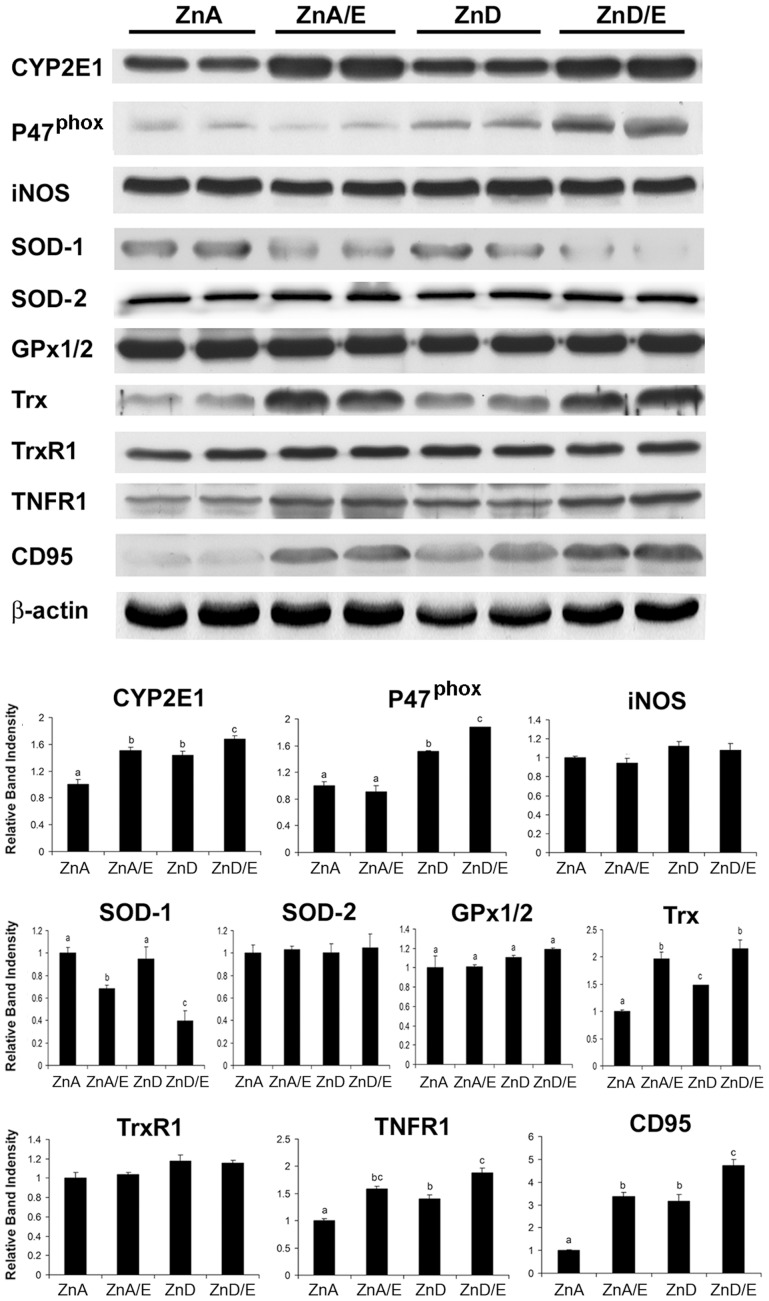
Protein levels of pro-oxidant and antioxidant enzymes and cell death receptors in the liver of mice chronically fed ethanol with zinc adequate or zinc deficient diet for 8 weeks. The immunoblot bands were quantified by densitometry analysis and the ratio to β-actin was calculated by setting the value of ZnA as one. Results are means ± SD (n4). Significant differences (*P*<0.05) between groups are determined by ANOVA. ZnA: zinc adequate diet. ZnA/E: zinc adequate diet plus ethanol. ZnD: zinc deficient diet. ZnD/E: zinc deficient diet plus ethanol.

The protein levels of hepatic pro-oxidant (CYP2E1, P47^phox^ and iNOS) and antioxidant enzymes (SOD-1, SOD-2, GPx1/2, Trx and TrxR1) and cell death receptors (TNFR1 and CD95) were determined by immunoblot, and the results are presented in Figure 6. CYP2E1 protein was increased in both ZnA/E and ZnD/E groups. The protein level of P47^phox^, a subunit of NADPH oxidase, was increased in ZnD and ZnD/E groups, particularly the latter. SOD-1 (Cu/Zn-SOD) protein was reduced in ZnA/E group, and a further reduction was found in ZnD/E group. Trx protein was remarkably increased in ZnA/E and ZnD/E group and moderately in ZnD group. The protein levels of TNFR1 and CD95 were increased in ZnA/E, ZnD and ZnD/E groups with a highest value in ZnD/E group. The protein levels of iNOS, SOD-2 (Mn-SOD), GPx1/2 and TrxR1 were not affected by either ethanol consumption or dietary zinc deficiency.

### Alterations of eWAT, Plasma Leptin Level and Hepatic Leptin Receptor

**Figure 7:**
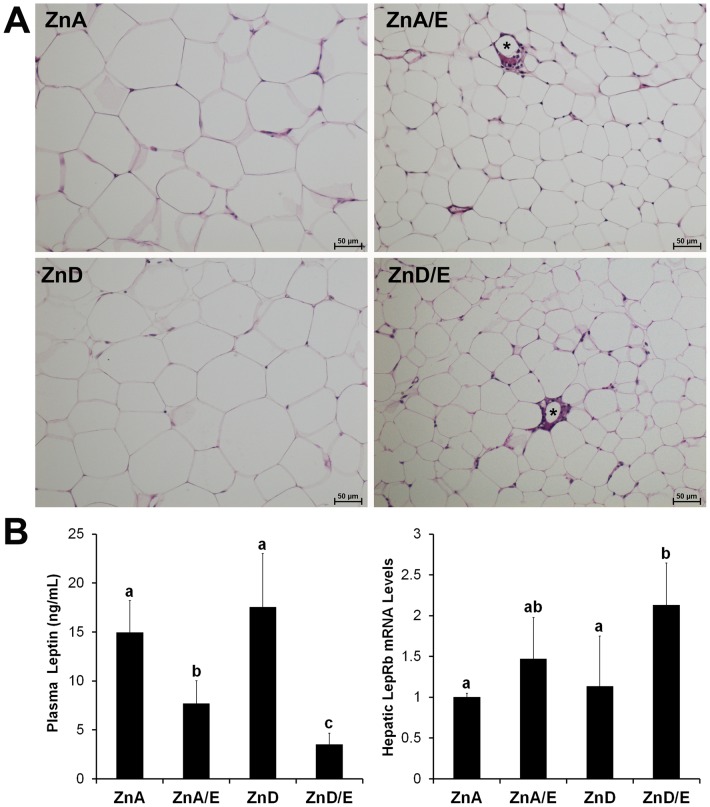
Alterations of epididymal white adipose tissue (eWAT) and leptin secretion in mice chronically fed ethanol with zinc adequate or zinc deficient diet for 8 weeks. **A:** Histopathology of eWAT. H&E staining. Stars: crown-like structures indicating degenerating adipocytes surrounded by inflammatory cells. Scale car50 µm. **B:** Plasma leptin level and hepatic leptin receptor (LepRb) expression. Plasma leptin levels were measured by an ELISA kit. Hepatic LepRb was detected by qPCR setting the value of ZnA as one. Results are means ± SD (n8–10 for plasma leptin level; n = 6 for hepatic LepRb). Significant differences (*P*<0.05, ANOVA) are identified by different letters. ZnA: zinc adequate diet. ZnA/E: zinc adequate diet plus ethanol. ZnD: zinc deficient diet. ZnD/E: zinc deficient diet plus ethanol.

Quantitative analysis of light microscopy images of eWAT (Figure 7A) demonstrated that the average diameters of adipocytes were 97.61±4.24 µm for ZnA group, 48.60±3.25 µm for ZnA/E group, 94.19±3.38 µm for ZnD group and 52.89±4.75 µm for ZnD/E group, respectively. Ethanol feeding reduced the size of adipocytes compared to ZnA or ZnD group regardless of the dietary zinc status. Crown-like structures, degenerating adipocytes surrounded with inflammatory cells, were also found in ZnA/E and ZnD/E groups. Zinc deficiency alone did not cause histopathological changes in the eWAT. The plasma level of leptin, one of the major adipokines, was reduced in ZnA/E group, and a further reduction was found in ZnD/E group. On the other hand, hepatic expression of leptin receptor, LepRb, was up-regulated in ZnD/E group in response to the decline of plasma leptin level (Figure 7B).

### Gut Permeability and the Development of Endotoxemia

**Figure 8:**
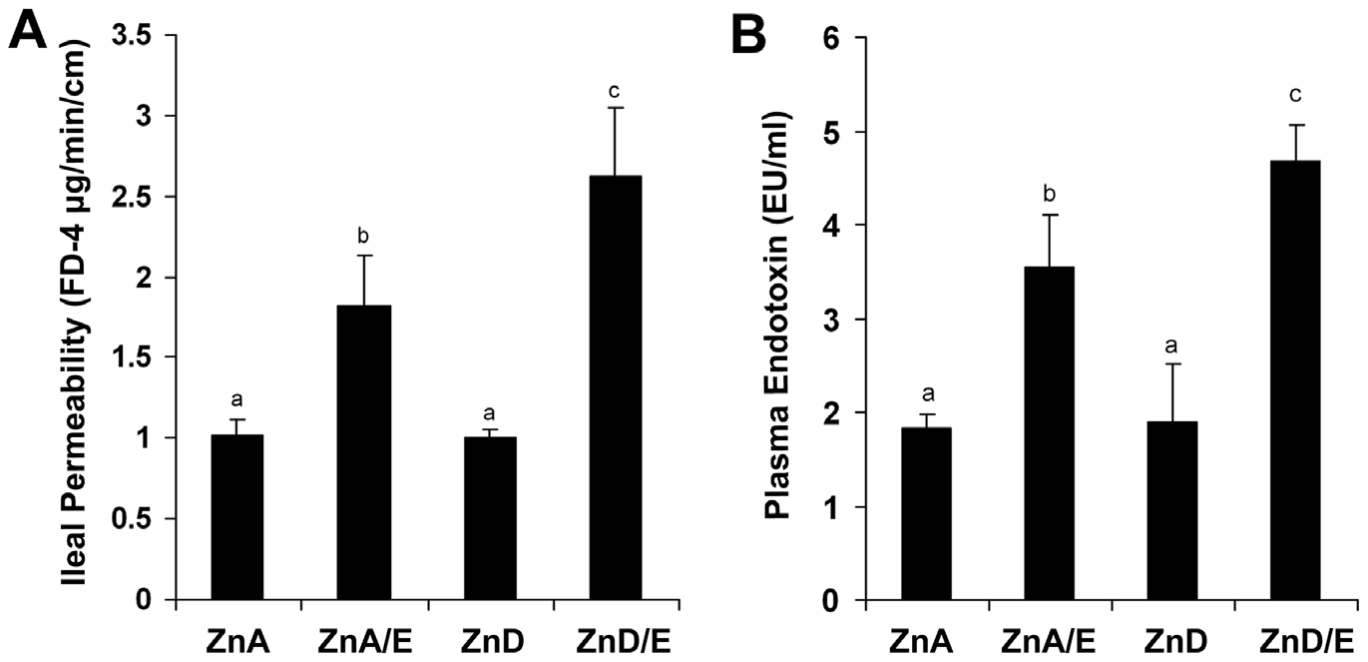
Gut permeability and plasma endotoxin in mice chronically fed ethanol with zinc adequate or zinc deficient diet for 8 weeks. **A:** Ileal permeability. The penetration of intralumen FITC-dextran (FD-4) to the incubation buffer was determined after incubation of the ileum sac for 20 minutes. **B:** Plasma endotoxin. Endotoxin levels were assayed by the limulus ameobocyte lysate (LAL) method. Results are means ± SD (n8–10). Significant differences (*P*<0.05, ANOVA) are identified by different letters. ZnA: zinc adequate diet. ZnA/E: zinc adequate diet plus ethanol. ZnD: zinc deficient diet. ZnD/E: zinc deficient diet plus ethanol.

Gut permeability to macromolecules were assessed by *ex vivo* measurement of ileal penetration of FD-4. As shown in Figure 8A, the ileal permeability to FD-4 was increased in ZnA/E group compared to ZnA group, and a further increase was found in ZnD/E group. In accordance with the increased gut permeability, the plasma endotoxin level was elevated in both ZnA/E group and ZnD/E groups with a greater value in the latter (Figure 8B). Zinc deficiency alone did not affect gut permeability and plasma endotoxin level.

## Discussion

The present study demonstrated interactions of dietary zinc deficiency and ethanol exposure in induction of liver injury including lipid accumulation and inflammation. Synergistic effects of dietary zinc deficiency on ethanol-induced oxidative stress in the liver were associated with an increased imbalance between pro-oxidative and antioxidant enzymes, particularly, the increase of NADPH oxidase and decrease of SOD-1. Dietary zinc deficiency also exaggerated ethanol-induced up-regulation of cell death receptors. Dietary zinc deficiency alone caused hepatic lipid accumulation but did not induce cytokine gene expression and neutrophil infiltration. Dietary zinc deficiency exaggerated ethanol-induced reduction of plasma leptin, although it did not further worsen ethanol-induced WAT mass reduction. Ethanol-induced gut permeability increase and plasma endotoxin elevation were exaggerated by dietary zinc deficiency. These data demonstrated, for the first time, that dietary zinc deficiency interacts with ethanol in the induction of liver injury.

Fatty liver is the earliest pathological change in the progression of alcoholic liver disease, and dysregulation of multiple lipid metabolic pathways may contribute to the development of alcoholic fatty liver, including up-regulation of fatty acid uptake and *de novo* lipogenesis and down-regulation of fatty acid oxidation and VLDL secretion [34]. Zinc has been shown to modulate hepatic gene expression and lipid homeostasis [25], [35], [36]. Alcohol consumption reduces hepatic zinc level, which may impair the function of zinc proteins [25], [37], [38]. Zinc finger transcription factors, peroxisome proliferation activator receptor-α (PPAR-α) and hepatocyte nuclear factor-4α (HNF-4α), play important roles in regulation of hepatic lipid metabolism. Our previous study demonstrated that zinc supplementation restored ethanol-inactivated PPAR-α and HNF-4α, leading to attenuation of alcoholic fatty liver [25]. Because clinical studies showed that alcoholics have a relatively lower zinc intake [27], [28], the present study utilized a marginal zinc deficient diet to test the possible interactions between dietary zinc deficiency and ethanol in induction of alcoholic fatty liver. As expected, zinc deficiency alone increased hepatic triglyceride, cholesterol and FFA levels in association with down-regulation of PPAR-α and lipid metabolism genes including Acsl1, Acox1 and Apob. These data suggest an important role of dietary zinc deficiency in lipid metabolic disorder and the development of alcoholic fatty liver. Interestingly, hepatic concentration of FFAs was increased only by dietary zinc deficiency. In accordance, hepatic expression of Acsl1 (acetyl-CoA synthetase long-chain family member 1), a gene responsible for fatty acid activation was down-regulated only by dietary zinc deficiency. Hepatic accumulation of FFAs has been shown to directly induce lipotoxicity or indirectly generate toxic byproducts such as ceramide and lysophosphatidylcholine [39]–[41]. Therefore, dietary zinc deficiency may contribute to the pathogenesis of alcohol liver disease through impairing lipid homeostasis as well as inducing lipotoxicity.

WAT plays a critical role in whole body energy homeostasis. It stores excess energy as triglyceride at positive energy balance condition, and releases fatty acids via lipolysis for usage by other organ system at negative energy balance conditions. However, WAT may release excess fatty acids at disease conditions, leading to ectopic lipid storage including fatty liver [7], [42]. WAT also regulates lipid metabolism in other organ systems by secreting adiponectin and leptin [43]. In the liver, adiponectin and leptin negatively regulate lipid content by stimulating fatty acid oxidation [44]. Previous studies have shown that ethanol exposure increases fatty acid uptake by hepatocytes [45]. Our recent study demonstrated that ethanol exposure stimulates WAT lipolysis which leads to a reduction of WAT mass in mice [7], [14]. By labeling triglycerides with deuterium, we found that ethanol exposure increased hepatic deposition of triglycerides which were stored in the WAT prior to ethanol exposure [7], [14]. Furthermore, we also demonstrated that ethanol exposure dramatically reduces the plasma leptin levels in association with reduction of WAT mass in mice [15]. Because one of the major roles of leptin is to stimulate energy expenditure, we tested whether leptin deficiency contributes to ethanol-impaired fatty acid oxidation in the liver. Indeed, normalization of plasma leptin level by administration of exogenous leptin stimulated fatty acid oxidation and attenuated alcoholic fatty liver in mice [15]. The present study did not find detrimental effect of zinc deficiency alone on the WAT. However, dietary zinc deficiency worsened ethanol-induced decline of plasma leptin level, which may contribute to the detrimental effect of zinc deficiency on ethanol-impaired hepatic fatty acid oxidation.

Hepatic cytokine production is a causal factor in ethanol-induced inflammation and hepatitis. Previous reports have shown that endotoxin is a trigger of cytokine production by Kupffer cells and hepatocytes [19], [46]. Our previous study demonstrated that experimental zinc deprivation induces IL-8 production in hepatoma cells, and histone hyper-acetylation due to inactivation of histone deacetylases is an epigenetic mechanism underlying the effect of zinc deprivation [47]. The present study demonstrated that dietary zinc deficiency alone did not affect hepatic cytokine genes and hepatic neutrophil infiltration. However, IP-10 and IL-1b were up-regulated only in the ZnD/E group, suggesting an interaction between zinc deficiency and ethanol in cytokine expression. Although zinc deficiency did not exaggerate ethanol-induced KC expression in the liver, it further elevated plasma KC level compared to ethanol alone, suggesting an interaction between zinc deficiency and ethanol in induction of whole body inflammatory response. Our previous studies with mouse models of acute and chronic ethanol exposure have shown that zinc impacts intestinal barrier function, thereby modulating endotoxin-induced hepatic cytokine production [9]. The present study further demonstrated that dietary zinc deficiency worsened ethanol-increased gut permeability and plasma endotoxin level. Moreover, endotoxin entered the bloodstream from intestinal lumen due to gut leakiness may elicit pro-inflammatory cytokine productions, which further impair the intestinal barrier [48], [49]. These data suggest that elevation of gut permeability due to interaction of dietary zinc deficiency and ethanol is an early event in the development of ethanol-induced hepatic inflammation.

Zinc has antioxidant properties, and dietary zinc levels impact hepatic antioxidant systems [50], [51]. Dietary zinc deficiency has been shown to induce hepatic lipid peroxidation in association with reduction of antioxidant enzymes such as SOD-1 [22], [23], [52]. The present study demonstrated dietary zinc deficiency and ethanol synergistically increased hepatic TBARS level, although zinc deficiency alone did not induce significant lipid peroxidation. Up-regulation of p47^phox^ and down-regulation of SOD-1 proteins were the major synergistic effects of zinc deficiency and ethanol on hepatic pro-oxidant and antioxidant systems. Zinc deficiency alone increased hepatic protein levels of CYP2E1 and p47^phox^ as well as cell death receptors, TNFR1 and CD95. These data indicate that zinc deficiency worsened ethanol-induced imbalance between pro-oxidant and antioxidant systems; this cellular disorder may provide a basic mechanism underlying the interactions of zinc deficiency and ethanol in dysregulation of lipid metabolism and inflammatory response in the liver.

In conclusion, the present study demonstrated that dietary zinc deficiency exacerbated ethanol-induced liver injury. Dietary zinc deficiency alone caused hepatic lipid accumulation, but did not affect hepatic inflammatory cytokine expression and neutrophil infiltration. Dietary zinc deficiency exaggerated ethanol-induced hepatic lipid accumulation and inflammatory response through modulating hepatic multiple lipid metabolism pathways and proinflammatory cytokine expression, respectively. While reduction of plasma leptin level is likely a factor mediating the interaction of zinc deficiency and ethanol in induction of fatty liver, elevation of gut permeability and plasma endotoxin level may account for the synergistic action in induction of hepatic inflammation. These data suggest that dietary zinc deficiency is a risk factor in alcoholic liver disease, and the synergistic effects of zinc deficiency on ethanol-induced pathogenesis involve multiple intrahepatic and extrahepatic factors, which also implicates the potency of zinc supplementation in ameliorating and/or preventing ALD.

## Supporting Information

Figure S1 **Hepatic malondialdehyde (MDA) accumulation.** Tissue distribution of MDA was detected by immunohistochemistry. CV: central vein. PV: portal vein. Scale car = 50 µm. ZnA: zinc adequate diet. ZnA/E: zinc adequate diet plus ethanol. ZnD: zinc deficient diet. ZnD/E: zinc deficient diet plus ethanol.
(TIF)
