# Supplementary figures and images for: Dietary Zinc Deficiency Exaggerates Ethanol-Induced Liver Injury in Mice: Involvement of Intrahepatic and Extrahepatic Factors
Source: PLoS One. 2013 Oct 14;8(10):e76522. doi: 10.1371/journal.pone.0076522 (PMC3796541; doi:10.1371/journal.pone.0076522)

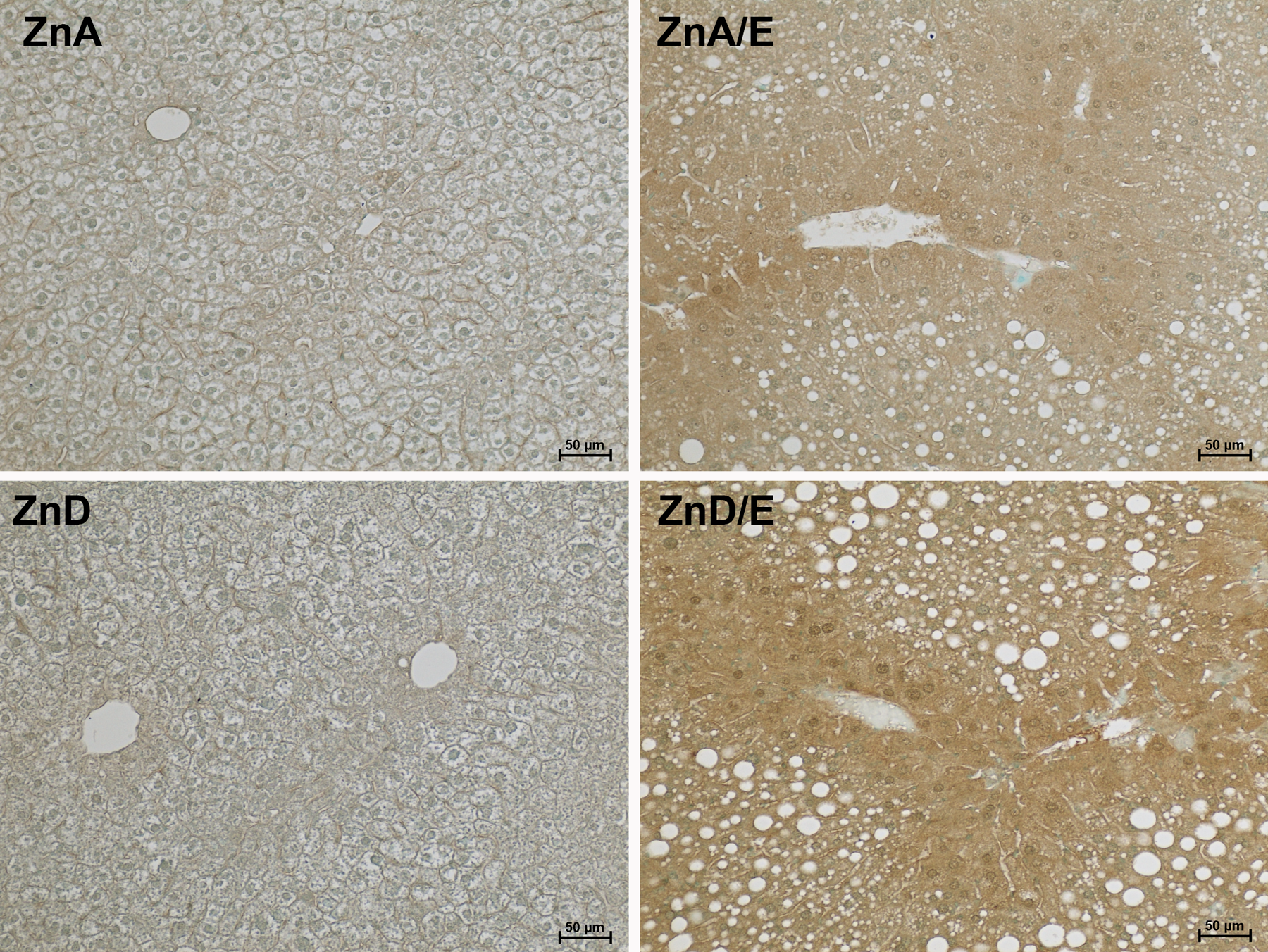

Supplement: Figure S1 — Hepatic malondialdehyde (MDA) accumulation. Tissue distribution of MDA was detected by immunohistochemistry. CV: central vein. PV: portal vein. Scale car = 50 µm. ZnA: zinc adequate diet. ZnA/E: zinc adequate diet plus ethanol. ZnD: zinc deficient diet. ZnD/E: zinc deficient diet plus ethanol. (TIF) [file pone.0076522.s001.tif]
